# Interventions to Increase Blood Donation among Ethnic/Racial Minorities: A Systematic Review

**DOI:** 10.1155/2019/6810959

**Published:** 2019-04-15

**Authors:** Jennifer K. Makin, Kate L. Francis, Michael J. Polonsky, Andre M. N. Renzaho

**Affiliations:** ^1^Menzies Institute for Medical Research, University of Tasmania, Hobart 7000, Australia; ^2^Murdoch Children's Research Institute, Melbourne 3052, Australia; ^3^Deakin Business School, Deakin University, Melbourne 3125, Australia; ^4^School of Social Sciences and Psychology, Western Sydney University, Sydney 2751, Australia; ^5^Translational Health Research Institute, Western Sydney University, Sydney 2751, Australia

## Abstract

Ethnic/racial minorities are under-represented in blood donor populations in most developed countries. This is of particular concern where minorities differ from a country's majority population in terms of blood or tissue typing, especially where type matching is required for effective management of rare disorders such as sickle-cell disease that require multiple transfusions. This systematic review assessed the effectiveness of interventions to increase blood donation among ethnic/racial minority populations in developed countries. We searched MEDLINE, EMBASE, CINAHL, and ProQuest on 20 March 2017 with no date restrictions and supplemented this with searches on Google Scholar, blood collection agency websites, reference lists of included studies, and a forward search of citations of included studies. We included intervention studies designed to increase recruitment and/or retention of adult, ethnic/racial minority blood donors in developed countries. The review identified eight studies reported in nine publications. Six were conducted in the USA with African Americans. Four studies reported on multifaceted, community-based interventions; three reported on one-off information and educational video interventions, presented face-to-face, or delivered via post or e-mail. The level of evidence for efficacy was low, and the majority of studies were assessed as having some risk of bias related to one or more methodological issues. All eight studies reported positive outcomes in blood donation and/or intention to donate. Seven trials found that the intervention increased presentation for donation, and three found an increase in the percentage of new donors from the ethnic minority targeted. The review findings demonstrate that it is possible to design and implement effective interventions to motivate individuals from ethnic/racial minority groups to donate blood. One-off interventions may be as effective as multifaceted, community-based interventions. There was insufficient evidence to recommend particular interventions, and future research should empirically assess alternative interventions using robust study designs.

## 1. Introduction

There is an acknowledged under-representation of ethnic/racial minorities in blood donor populations in many if not most developed countries, despite otherwise well-established voluntary blood collection systems in these countries [1]. This issue is increasingly important given the growing multicultural nature of communities, partly arising from the increased numbers of refugees relocating from developing to developed countries [2]. For example, nearly half of all Australians in 2016 were either born in another country or had at least one parent who was born in another country [3]. In the USA, population projections show continued increases in ethnic/racial minority proportions, rising to 28.6% Hispanic or Latino and 14.3% black or African American by 2060 [4].

Efforts to increase representation of ethnic/racial minorities in blood donor populations are important for three reasons [1]:

1. Individuals from some minority groups may differ from a country's majority population in terms of extended blood or tissue typing. This under-representation of rare blood types is of particular concern in ensuring appropriate blood supplies to avoid alloimmunisation and effectively manage conditions, such as sickle-cell disease, which require frequent transfusions and are more common among certain ethnic/racial minority populations [5] (although it has recently been noted that in the USA, African Americans do not supply the majority of multiple antigen negative units [6]).
2. With demographics shifting towards an increase in individuals from different ethnic/racial minority populations, the assembly of a large group of potential new donors arises, which is important for ensuring adequate overall blood supply.
3. Participating in blood donation may facilitate the integration of ethnic/racial minority populations to the country's healthcare system, thereby contributing to a reduction in health inequities for these populations [7].

However, ethnic/racial minorities encounter additional barriers to donation [8], such as: a lack of citizenship or national ID cards, higher deferral/exclusion rates (e.g., deferral rates due to low haemoglobin have been estimated to be two to three times higher among African Americans compared with White Americans), socioeconomic and sociocultural issues, religious beliefs, myths about blood donation, knowledge gaps, lack of trust between minorities and the blood donation services, a fragmented and hard to reach target audience, organisational paradigms and inertia, and language barriers which include problems recruiting and maintaining bilingual staff [1, 9–11]. Although some of these barriers may be common among multiple ethnic/racial groups, some minority groups encounter specific barriers due to their cultural or historical context. Therefore, tailored interventions that focus on barriers specific to certain ethnic/racial minority groups may be needed to recruit and retain blood donors from these groups, as was recommended by the Review of Australia's Plasma Fractionation Arrangements in 2006 [12]. This is also suggested by the research of the Missing Minorities group of the European Blood Alliance into blood donation services within the 23 countries participating in their research [1]. Targeting interventions for ethnic/racial minorities is also discussed in growing literature examining culturally competent or adapted health interventions and services [13, 14].

Five types of strategies for tailoring interventions for particular cultural groups have been identified [14]:

1. Peripheral: ensuring interventions appear culturally relevant, for example through pictures, colours, or headings specifying the group.
2. Evidential: using evidence specific to the cultural group to enhance perceived relevance.
3. Linguistic: ensuring language used is culturally tailored.
4. Constituent-involving: having substantive roles in intervention delivery for members of the cultural group.
5. Sociocultural: incorporating the broader social and cultural characteristics of the group into intervention design and delivery.

Although there is some evidence for the effectiveness of culturally tailoring healthcare interventions to increase access and utilisation of blood donation and other health services, evidence for particular strategies is limited [15].

Interventions to recruit and retain blood donors in the general population have been classified into five approaches: motivational, reminders/asking, measurement of cognitions, incentives, and preventing vasovagal reactions (see Table 1). Effective recruitment and retention are two different processes and may require different approaches. For example, retention may be influenced more by interventions that focus on actions during or after donation rather than interventions prior to the blood donation appointment.

Globally, a variety of strategies to specifically target minority donors have been reported by several national blood donation organisations, including media campaigns (using traditional and newer forms of media, such as social media), interventions through minority and/or religious organisations, increased minority staff in blood donation organisations, health practitioner-led interventions, donor-recruits-donor interventions, public event recruitment, door-to-door recruitment, and public health initiatives such as cardiovascular screening [1, 17, 18]. These mirror the variety of targeted proactive and reactive strategies found to be effective in recruiting minority individuals to health research, although evidence is lacking to recommend one strategy over another [19].

Research has suggested that there have been some small improvements in donor diversity; however, it is unclear whether these are the result of targeted interventions or due to demographic changes in the various communities. For example, in the USA, while the proportion of nonwhite donors increased in the decade from 2006–2015, this essentially paralleled their relative increase in the population, making it difficult to draw conclusions regarding the effectiveness of targeted recruitment campaigns implemented over the same period [4]. A recent survey-based study describing the current state of minority blood donor recruitment in 23 countries concluded that while there is a great awareness of the under-representation of migrant communities, the implementation of targeted recruitment and retention strategies are at a very early stage of development in many of these countries [1]. While papers have been written evaluating recruitment of specific minorities for blood donation in given countries, to date there is no integrative review comparing the strategies used in these studies to engage with migrant communities across contexts [20].

In response to this gap in the literature, as well as to inform future interventions and research, this systematic review aimed to answer the following question: how effective are interventions conducted to increase blood donation among ethnic/racial minority populations in developed countries? The literature search aimed to identify intervention studies designed to increase blood donation recruitment and/or retention, regardless of study design that included adult participants with an ethnic/racial minority background living in high income countries and that reported on any outcome measure of blood donation behaviour or intentions.

## 2. Methods

The review was registered on Prospero, the international prospective register of systematic reviews, prior to commencement in March 2017 (no. CRD42017058919).

### 2.1. Searches

MEDLINE, EMBASE, CINAHL, and ProQuest databases were searched on 20 March 2017. The search combined terms related to (1) blood donation, and (2) ethnic/racial minorities. The search was kept deliberately wide by not including terms attempting to limit the results to intervention studies or to adult participants from particular countries. Instead, noneligible studies were removed from the resulting broader pool of articles during screening. A full list of MeSH terms and text words used can be found listed in Table S1, in the Supplementary Material. No date or language restrictions were applied to the search; materials published as comments, letters, editorials, or news were excluded.

The first 100 pages (i.e., 1,000 references) from a Google Scholar search were screened for additional potentially eligible articles not identified in the database search. Additionally, the websites of the main blood collection agencies in Australia [21], New Zealand [22], Canada [23], and the USA [24, 25] were searched for published articles and intervention reports, as was that of the European Blood Alliance [26]. The “Missing Minorities” group of the European Blood Alliance was also approached to obtain a copy of their extensive list of available literature [20]. Furthermore, reference lists of articles meeting inclusion criteria were reviewed and a forward search of articles that cited each of the included articles was undertaken to identify additional articles that met the inclusion criteria.

### 2.2. Study Inclusion Criteria

To be included, studies needed to meet all four eligibility criteria listed in Table 2.

Studies reported only in conference abstracts were excluded as these lacked sufficient detail on interventions and outcomes. However, the authors of seven conference abstracts reporting on potentially eligible studies were contacted and copies of any subsequent publications were requested, but none were provided.

All references were downloaded to EndNote. Duplicates were removed, and then titles and abstracts were screened against the eligibility criteria. Full text articles for potentially relevant references were reviewed independently by two authors.

Among the articles identified as eligible through this process were four review articles [28–31]. These review articles were broader in focus than the current review and none was based on a systematic literature search with predefined eligibility criteria. An examination of the references for the four reviews did not identify any additional eligible studies.

### 2.3. Study Quality Assessment

Quality was assessed for descriptive purposes rather than to inform study inclusion or exclusion. Two authors critically appraised each of the included studies using a standardised appraisal form, which included NHMRC (National Health and Medical Research Council) level of evidence and magnitude of effect rating [32], and risk of bias was assessed using CASP tools [33] (see Table S2 in the Supplementary Material for definitions and full description of risk of bias domains). The form was initially trialled with two of the included articles, to ensure the two judges were in agreement on the method before proceeding to full extraction and appraisal. In only one case did a discrepancy in judgements exist, in regards to the magnitude of effect rating. The judges discussed their appraisal to reach a consensus.

### 2.4. Data Extraction

Two authors independently extracted relevant data as predefined in the registered protocol from intervention studies meeting the selection criteria (data extraction form available on request). This included study type and characteristics, participant characteristics, details of interventions, and details of comparison groups if present. Primary outcomes extracted were reported blood donation behaviour and blood donation intention. Secondary outcomes were attitudes towards blood donation. The data extraction form was piloted with two of the included articles to ensure the two authors understood and agreed on the process prior to extraction of data from the remaining studies.

### 2.5. Data Synthesis and Presentation

Outcomes were tabulated and summarised in a narrative synthesis of findings. The incomplete and inconsistent reporting of different outcome measures that could not be standardised across studies meant that the results could not be pooled in a quantitative meta-analysis.

## 3. Results

### 3.1. Review Statistics

**Figure 1:**
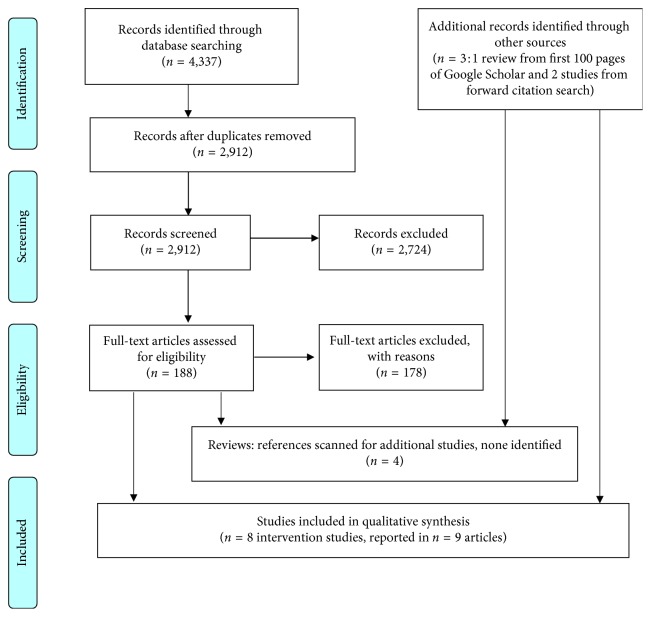
PRISMA flow diagram [43].

After removal of duplicates, 2,912 titles and abstracts were screened against the eligibility criteria, resulting in 188 potentially relevant references. Through review of full text articles for these references, eight intervention studies meeting the eligibility criteria were identified, reported in nine publications (one study was published in two different journals) [34–42]. PRISMA flow diagram is shown in Figure 1.

The most common reasons for exclusion of full text articles were (a) articles did not report on the evaluation of an intervention (95 articles) and (b) the studies were not conducted in a high income (i.e., developed) country (41 articles). The complete list of reasons for exclusion of full text articles is included in Table S2, within the Supplementary Material.

Table 3 shows the characteristics of the eight included studies. Six of the eight interventions were conducted in the USA with African Americans (two also included black and Hispanic or Latino individuals) [34–37, 40, 42], one in Canada with the Haitian community [38, 39], and one in France with the Comorian community [41]. Three studies targeted new donors [37, 40, 42], one targeted individuals who had previously donated [34], and three targeted both new and previous donors [35, 36, 38, 39].

Four studies reported on a one-off information or one-off education intervention. One trialled a face-to-face educational session and video [37], one was a mail-out of an educational video and brochures [35], and one sent information and a web link to a video via e-mail [34]. The remaining study tested a computer-tailored intervention based on the transtheoretical model [36]. The other four studies reported on multifaceted, community-based interventions [38–42]. These included a variety of repeated activities in target communities, most including a media component.

All interventions could be classified as using motivational (cognitions-based) techniques, according to Godin et al.'s typology of blood donation recruitment and retention interventions [16]. Some studies also reported using other motivational techniques: altruism [36, 37, 41, 42] and modelling [36]. Only two studies reported using additional types of intervention components: one reported using reminders [34] and one reported using measurement of cognitions and preventing vasovagal reactions [36]. All but one intervention used materials tailored for the minority group; the remaining study trialled and compared both tailored and nontailored versions of the intervention [34].

### 3.2. Study Quality Assessment

Table 4 presents the results of the critical appraisal. None of the interventions were evaluated using randomised controlled trials, and the overall level of evidence as determined by study design was low. All included studies were designated as NHMRC evidence level III-2 (nonrandomised experimental trials [35, 42] and cohort studies [34, 37]), III-3 (historical control studies [38–40]), or IV (case series [36, 41]). The majority of studies were assessed as having some risk of bias on one or more methodological issue, most commonly a failure to control for confounding factors, a lack of accuracy in measuring exposure to the intervention and/or outcomes, and/or issues with incomplete or short-term follow-up of participants.

### 3.3. Evidence of Effectiveness

All eight trials reported positive outcomes in blood donation and/or intention to donate, some with statistical comparisons with a control group or with a historical comparison period (Table 5). Seven trials found that the intervention increased presentation for donation; three found an increase in the percentage of new donors. Most trials focused on short-term outcomes (i.e., mainly measured immediately following the intervention) and thus the long-term impact of interventions is unclear.

One study tested the effectiveness of a recruitment e-mail and video link tailored specifically to African American potential donors, compared with generic versions of the same materials [34]. While both were effective in driving presentation for donation (see Table 5), they found no significant difference in the opening rate for the culturally tailored e-mail versus the generic e-mail (1905/9142: 20.8% vs. 1953/9147: 21.4%; *p*=0.39) and no significant difference in the donation presentation rate during the subsequent 76-day period (126/9142: 1.4% vs. 122/9147: 1.3%; *p*=0.79).

Two studies also reported on the impact on participants' attitudes to blood donation. Sutton [37] focused on the relationship between attitude and historical events, using responses on a five-point Likert scale to five items: (a) historical events, such as the Tuskegee experiment, make me nervous about donating blood; (b) the medical establishment cannot be trusted; (c) trust plays a major part in my decision to become a blood donor; (d) my prior knowledge about blood donation motivates me to donate blood; (e) knowledge of prior mistreatment of African Americans affects my decision to donate blood. No statistically significant difference in attitude was found before and after an education session *t* (146) = −1.455, *p*=0.148. Robbins et al. [36] assessed “Decisional Balance,” using a 12-item measure where participants rate “Pros” (e.g., saving someone's life), “Eligibility Cons” (e.g., find out I have a disease), and “Physical Cons” (e.g., afraid of needles) on a 5-point scale to reflect how important each item is in their decision whether or not to be a regular blood donor. They found a significant increase in reported “Physical Cons,” *t* (149) = 2.41, *p*=0.017, *d* = 0.20. No significant differences were found for “Pros” or “Eligibility Cons.” However, they also examined stage progression according to the transtheoretical model and found that 46.9% of those in a preaction stage at pretest progressed at least one stage at posttest assessment.

## 4. Discussion

This systematic review of interventions targeting ethnic/racial minority blood donation behaviour found there was limited research to determine the effectiveness of interventions to increase blood donation among ethnic/racial minority populations in developed countries. While all eight interventions identified were reported to have positive effects on presentation for blood donation, the research designs of all the studies lacked robustness, with no randomised or pseudorandomised controlled trials identified. Additionally, sample sizes were not reported in four studies, and three did not report statistical comparisons with historical or concurrent controls. All studies were found to be at some risk of internal bias, principally due to failure to control for potential confounding factors and/or to accurately measure exposure to interventions. The consistently positive direction of results suggests some degree of publication bias may be operating. This finding is consistent with reviews of other health interventions, which suggest a generally positive bias in the publishing of results exists and that negative or insignificant results are less frequently published [44]. Despite this, the positive results reported by these trials suggest interventions can be effective in motivating individuals from ethnic/racial minority groups to donate blood.

Two main categories of intervention were documented: (a) relatively sustained multifaceted, community-based interventions and (b) one-off information and educational video interventions, presented face-to-face, or delivered via post or e-mail. There was no indication that either approach was more successful, despite the differences in scope and investment (although no economic evaluation occurred in any of the studies), suggesting that relatively simple targeted interventions relating to behavioural change and economic efficiency may be worth trialling. This is particularly important in a context where cost constraints often limit organisations' ability to implement or continue with donor recruitment or retention campaigns targeting specific population groups.

Evidence was lacking to inform the selection of intervention type, according to the typology developed by Godin et al. [16]. The interventions identified suggest that motivational, cognitions-based approaches could be effective, but there was little evidence to support the effectiveness of other intervention types. The fact that one study found there were limited changes in underlying views but that there was a movement along the transtheoretical model suggests that focusing solely on underlying views may in fact miss an important behaviour change.

Publication bias could result in ineffective intervention strategies being under-reported. It is also possible that the targeted literature search strategy used in this review may not have identified evaluations of other successful interventions which included individuals from ethnic/racial minorities within a broader population [18]. However, the much broader range of intervention types trialled among the general population merit consideration when designing interventions to increase blood donation recruitment and retention among ethnic/racial minorities.

Strategies to increase blood donor recruitment and retention among ethnic/racial minorities may include tailoring materials to the specific group and/or targeting the group by ensuring the campaign reaches them [14]. Only one study compared culturally tailored and nontailored recruitment campaign materials, and it found tailoring had no effect on blood donation rates within the minority group [34]. The remaining interventions tailored educational and promotional materials to the ethnic/racial minority group using a variety of strategies, with positive outcomes. However, it is not possible to determine whether these were due to the effective tailoring of materials, or to the intervention successfully reaching the intended target group, as past research suggests that one reason ethnic groups may not engage with messages is simply because they do not see them. For example, the Missing Minorities group of the European Blood Alliance, a collaboration between blood services in eleven countries initiated by the European Blood Alliance in 2012 [20], identified that in many cases, minority groups are not being reached by the general recruitment methods used by blood establishments. The project also identified overlapping motivators and barriers to blood donation among ethnic/racial minorities and the broader population, implying that, in some cases, simply ensuring that recruitment campaigns reach minority groups may improve blood donation rates. In cases where a particular minority is targeted for recruitment, it is good marketing practice to tailor the messaging and campaign materials accordingly, with more general calls for the adaption of health promotion and support when targeting culturally distinctive groups [45, 46]. However, in population-wide campaigns, messaging and materials should be designed to be inclusive of all population groups targeted.

Relative to studies of interventions to increase blood donation among ethnic/racial minorities, many more published studies of barriers and motivators to donation among these communities (e.g., [47–50]) and a recent systematic review [11] have been published. While theoretically valuable for the development of targeted interventions, very few published reports have been published of interventions that have been developed based on the findings of this formative research. While the circumstances of different ethnic/racial minorities are very specific, particular, and geographically local, many of the barriers and motivators have been previously studied and/or are common to multiple ethnic/racial minority groups [1] and may also impact on the wider community [8]. This suggests that new interventions could initially be developed using existing research, rather than repeating formative research in new populations. This is not to downplay the importance of engagement with and understanding of ethnic/racial minority groups when developing interventions; however, scarce research resources might be better directed to pilot testing and refining interventions using robust study designs, rather than further describing the target groups.

The Action Plan developed by the Missing Minorities group of the European Blood Alliance could act as a useful starting point for targeting activities [20]. The Action Plan steps through key considerations in developing an intervention to increase blood donation among ethnic/racial minorities, including data availability and collection by blood services and considering activities targeting both the target group and blood service staff. A systematic research agenda building on this Action Plan is warranted. Even if targeted recruitment of minorities to fulfil extended matching criteria for conditions such as sickle-cell disease is less crucial than previously assumed [6], increasing recruitment remains important to maintain the overall blood supply in a changing demographic context [1] and in promoting integration in the wider health system [7]. As a first step, this should include ensuring blood services routinely record ethnicity/race, to allow monitoring and evaluation of the effectiveness of interventions to increase recruitment and retention of ethnic/racial minorities. In designing intervention studies, care should be taken to overcome some of the more common sources of potential bias identified in this review. In particular, exposure to the intervention should be accurately recorded, a control group or time period should be included to allow statistical comparisons, and studies of longer duration to assess sustainability of positive effects should be considered.

## 5. Conclusions

In conclusion, the results of this systematic review suggest that interventions aiming to increase blood donation among ethnic/racial minorities can be effective. Broadening participation in blood donation would have the advantage of increasing the overall blood supplies, as well as improving the ability to match rare blood types where required [41]. Including ethnic/racial minorities in blood drives has the added advantage of promoting increased inclusion and participation in the broader community [7]. Given the lack of high quality intervention trials in the literature, these should be a priority for future research efforts, especially in multicultural countries where mass messages may not resonate with ethnic/racial minority communities.

## Figures and Tables

**Table 1:** Classification of blood donation recruitment and retention interventions (adapted from Godin et al. [16]).

Type of intervention	Definition
Motivational	Interventions aimed at increasing motivation toward blood donation
Cognitions-based	Interventions targeting psychosocial cognitions related to motivation, such as social norms, attitudes, and barriers
Foot-in-the door/door-in-the-face	Interventions using the foot-in-the-door, the door-in-the-face, or a combination of both techniques to motivate individuals to give blood. Foot-in-the-door involves asking a small request that should be accepted and then asking a critical large request. Door-in-the-face involves asking a large request that should be refused and then asking a critical small request
Altruism	Interventions using altruistic motives to motivate individuals to give blood
Modelling	Interventions showing another person giving blood to motivate individuals to give blood

Reminders/asking	Interventions using direct requests or reminders about the next eligibility date and/or the next appointment to give blood (e.g., telephone call prompt)

Measurement of cognitions	Interventions using the completion of a questionnaire about the intention to give blood to activate cognitions about blood donation (e.g., question-behaviour effect)

Incentives	Interventions using incentives for donating blood such as a T-shirt, money, prizes, tickets, and other

Preventing vasovagal reactions	Interventions to avoid dizziness and fainting, including applied muscle tension during donation, predonation salt loading, on-site stomach distension with liquids, donor distraction techniques, more stringent estimated blood volume requirements for donors under age 23, salty postdonation snacks, intensive education for individuals with higher fear scores

**Table 2:** Study inclusion criteria.

	Inclusion criterion
1	Evaluate an intervention designed to increase blood donation recruitment and/or retention, regardless of study design

2	Include adult participants in a country classified as high income by the World Bank [27] (as a proxy indicator of a formalised blood donation and supply system) [11]

3	Include participants with an ethnic/racial minority background that:
	(a) was important to blood or tissue typing and/or
	(b) who formed a large group of potential new donors [1] and/or
	(c) were from a different country/region of origin to the majority of a country's population and/or
	(d) differed linguistically from the majority language of a country

4	Report on any outcome measure of blood donation behaviour or blood donation intentions

**Table 3:** Study characteristics.

Authors	Study type	Country	Ethnic/racial group	Sample size	Previous/new donors	Intervention type	Intervention	Tailoring	Comparison	Outcomes
*One-off information and education interventions*										
Bachegowda et al. [34]	Cohort	USA (New York)	African American	*n* = 18,638	Previous	Motivational (cognitions-based); reminders.	Two e-mails, one culturally tailored, one not. Included web link to video.	Peripheral (e-mail subject specified more African Americans need blood transfusions; video provided testimonial from African American woman with SCD); evidential (content presented the need to address the burden of sickle-cell disease (SCD))	Not opening e-mail	Primary: return presentation for donation
Price et al. [35]	Nonrandomised, experimental trial with historical control	USA (Missouri, St. Louis)	African American	*N* = 5,000 (mail-out), *n* = 176 (survey)	Previous/new	Motivational (cognitions-based)	Mail-out to approx. 50% of households of an introductory postcard, educational video and brochures.	Peripheral (use of African American recording artist on postcard and video); sociocultural (video addressed barriers to blood donation among African Americans identified in previous project)	(1) 6 month interval from the previous year; (2) geographically adjoining zip codes	Primary: number of presenting donors
Robbins et al. [36]	Case series	USA (eight states in the northeast region)	Black (10.7% Hispanic/Latino)	*n* = 150	Majority (76.7%) previous	Motivational (cognitions-based, altruism, modelling); measurement of cognitions; preventing vasovagal reactions.	Computer-tailored intervention based on the transtheoretical model (TTM) accessed via the Internet. Intervention components included testimonials, images and graphics, behaviour change strategies, and feedback sections.	Evidential (blood donation in the context of SCD); not further specified	None	Primary: likelihood of considering donating blood Secondary: stage of change progression, change in attitudinal “pros” and “cons.”
Sutton [37]	Prospective cohort	USA (Virginia)	African American	*n* = 155 (*n* = 124 in analysis)	New	Motivational (cognitions-based, altruism)	Educational session, including a researcher-led lecture, sickle-cell video, question and answer period, and social media.	Evidential (education on importance and uses of blood donated by African Americans, video provided testimonial from African Americans); sociocultural (addressing barriers identified in the literature).	African American population of the area as a whole	Primary: attempt to donate blood Secondary: attitudes
*Multifaceted, community-based interventions*										
Charbonneau and Daigneault, [38]; Charbonneau and Tran, [39]	Historical control	Canada (Quebec)	Black/Haitian	Not specified	Previous/new	Motivational (cognitions-based)	53 outreach activities (information booths, targeted presentations, group discussions, participation and sponsoring of community and cultural events, forums with leaders, radio interviews, targeted marketing, tours of laboratories, 27 blood drives)	Peripheral (sponsoring of community event); evidential (raising awareness of SCD); constituent-involving (organised blood drives in collaboration with community associations, direct requests from individuals with SCD); not further specified	None	Primary: number of black community donors
Frye et al. [40]	Historical control	USA (New York)	African American or black and Hispanic or Latino	Not specified	New	Motivational (cognitions-based)	Outreach coordinators: Included outreach to key leaders and companies; presentations at events; educational presentations at educational, civic, religious and community-based organisations. Tailored marketing materials in both English and Spanish. Newspaper coverage and radio commercials.	Peripheral (images of racially and ethnically diverse communities); evidential (critical need for African Americans to donate blood); constituent-involving (building partnerships with communities and their leaders); sociocultural (addressing barriers identified in qualitative research)	Donors recruited through a drive with the same population, before the involvement of the coordinator.	Primary: units of blood in 14 months from African American and Hispanic or Latino American
Grassineau et al. [41]	Case series	France (Marseilles)	Comorian community	Not specified	Not specified	Motivational (cognitions-based, altruism)	Community action group for voluntary blood donors, including local media and community meetings.	Evidential (specific importance for Comorian community); Linguistic (Comorian-speaking local radio); constituent-involving (community action group, involvement of religious and political leaders); sociocultural (explaining elements of Western culture to the Comorian community, addressing barriers identified through research)	None	Primary: volunteering for blood donation
Price et al. [42]	Non-randomised, experimental trial	USA (Missouri, St. Louis)	African American	Reach: approx. 15,000 people	New	Motivational (cognitions-based, altruism)	34 blood drives at 13 churches, including education session at church.	Constituent-involving (African American church sponsorship of blood drive, presented by community members); not further specified	Donors in the general population	Primary: percentage of first-time blood donors

**Table 4:** Critical appraisal.

	NHMRC level of evidence	Magnitude of effect	Clearly focused issue	Recruitment acceptable	Exposure accurately measured	Outcome accurately measured	Confounding factors identified	Confounding factors controlled	Follow-up—complete enough	Follow-up—long enough	Total acceptable
Bachegowda et al. [34]	III-2	High	+	+	+	+	?	−	+	+	6/8
Charbonneau and Daigneault [38]; Charbonneau and Tran [39]	III-3	High^*∗*^	+	+	−	?	−	−	?	+	3/8
Frye et al. [40]	III-3	High^*∗*^	+	+	+	+	+	+	+	+	8/8
Grassineau et al. [41]	IV	Med^*∗*^	+	+	−	+	−	−	+	?	4/8
Price et al. [35]	III-2	High	+	+	+	+	+	+	+	+	8/8
Price et al. [42]	III-2	High	+	+	+	+	+	+	+	+	8/8
Robbins et al. [36]	IV	Med	+	+	+	+	+	+	+	−	7/8
Sutton [37]	III-2	High	+	−	+	−	+	−	−	+	4/8

^*∗*^Judgement based only on absolute increases in donor numbers as no statistical comparison was reported.

**Table 5:** Primary intervention outcomes.

Study (intervention)	Primary outcome(s)	Follow-up	Number/proportion of donors	% New donors
*One-off information and education interventions*				
Bachegowda et al. [34] (emailed information and video link)	Return presentation for donation	76 days	2.5% of those who opened the e-mail presented for blood donation, compared with 1.0% of those who did not open the e-mail (*p* < 0.001).	NA

Price et al. [35] (mailed information and video)	Number of presenting donors	6 months	75% (217 vs. 124) increase (*p*=0.05) compared to first 6-month interval from previous year. No significant increases in geographically adjoining zip codes during the same period.	64% (126 vs. 77) increase (*p*=0.02) compared to previous year.

Robbins et al. [36] (computer-tailored intervention)	Likelihood of considering donating blood	Immediate	More likely to consider after completing the program (*t* (149) = 3.56, *p*=0.001, *d* = 0.29).	

Sutton [37] (face-to-face information and video)	Attempt to donate blood	2 months	16% (*n* = 20/124), compared with 9% of African Americans in the area (*z* = 3.039, *p* < 0.001).	

*Multifaceted, community-based interventions*				
Charbonneau and Daigneault [38]; Charbonneau and Tran [39] (53 outreach activities)	Number of black community donors	NR	Increased from 170 in 2009 to 1,582 in 2012.	53%

Frye et al. [40] (outreach coordinators)	Units of blood from African American and Hispanic or Latino American	Up to 14 months (during active recruitment period)	Incremental increase of 1,574 African American and Hispanic or Latino American units. 15% were rare blood donors, compared with 4% of typical NYBC drives.	Nearly three-quarters, compared with 17% of NYBC drive donors.

Grassineau et al. [41] (community action group)	Volunteering for blood donation	Immediate	Total of 92 individuals of Comorian origin volunteered for blood donation (no reported comparison period, control group, or size of population exposed to intervention).	NR

Price et al. [42] (repeated blood drives at churches)	Percentage of first-time blood donors	Immediate	NA	60% (428 of 699), compared with 12.2% (21,516 of 175,818) for the area (*p*=0.001)

NA: not applicable; NR: not reported.

## Supplementary Materials

Supplementary Materials Supplementary Material file includes Tables S1 (search terms), S2 (criteria for critical appraisal), and S3 (reasons for exclusion of full-text articles).

## References

[1] van Dongen A., Mews M., Kort W. L. A. M. D., Wagenmans E. (2016). Missing minorities—a survey based description of the current state of minority blood donor recruitment across 23 countries. *Diversity and Equality in Health and Care*.

[2] OECD (2017). *International Migration Outlook 2017*.

[3] Australian Bureau of Statistics (2017). *2071.0—Census of Population and Housing: Reflecting Australia—Stories from the Census, 2016*.

[4] Yazer M. H., Delaney M., Germain M. (2017). Trends in US minority red blood cell unit donations. *Transfusion*.

[5] Shaz B. H., Zimring J. C., Demmons D. G., Hillyer C. D. (2008). Blood donation and blood transfusion: special considerations for African Americans. *Transfusion Medicine Reviews*.

[6] Yazer M. H., Anani W. Q., Denomme G. A. (2018). Trends in antigen-negative red blood cell distributions by racial or ethnic groups in the United States. *Transfusion*.

[7] Polonsky M. J., Ferdous A. S., Renzaho A. M. N., Waters N., McQuilten Z. (2017). Factors leading to health care exclusion among African refugees in Australia: the case of blood donation. *Journal of Public Policy & Marketing*.

[8] Bednall T. C., Bove L. L. (2011). Donating blood: a meta-analytic review of self-reported motivators and deterrents. *Transfusion Medicine Reviews*.

[9] Polonsky M., Francis K., Renzaho A. (2015). Is removing blood donation barriers a donation facilitator?. *Journal of Social Marketing*.

[10] Polonsky M. J., Renzaho A. M. N., Brijnath B. (2011). Barriers to blood donation in African communities in Australia: the role of home and host country culture and experience. *Transfusion*.

[11] Klinkenberg E. F., Huis In ’t Veld E. M. J., de Wit P. D. (2018). Blood donation barriers and facilitators of Sub-Saharan African migrants and minorities in Western high-income countries: a systematic review of the literature. *Transfusion Medicine*.

[12] Flood P. (2006). *Review of Australia’s Plasma Fractionation Arrangements*.

[13] Anderson L. M., Scrimshaw S. C., Fullilove M. T., Fielding J. E., Normand J. (2003). Culturally competent healthcare systems. *American Journal of Preventive Medicine*.

[14] Kreuter M. W., Lukwago S. N., Bucholtz D. C., Clark E. M., Sanders-Thompson V. (2003). Achieving cultural appropriateness in health promotion programs: targeted and tailored approaches. *Health Education & Behavior*.

[15] Truong M., Paradies Y., Priest N. (2014). Interventions to improve cultural competency in healthcare: a systematic review of reviews. *BMC Health Services Research*.

[16] Godin G., Vézina-Im L.-A., Bélanger-Gravel A., Amireault S. (2012). Efficacy of interventions promoting blood donation: a systematic review. *Transfusion Medicine Reviews*.

[17] France C. R., France J. L., Carlson B. W. (2017). Applying self-determination theory to the blood donation context: the blood donor competence, autonomy, and relatedness enhancement (blood donor care) trial. *Contemporary Clinical Trials*.

[18] Kessler D. A., Ortiz C., Grima K. (2012). Cardiovascular disease risk assessment and prevention in blood donors. *Transfusion*.

[19] Ibrahim S., Sidani S. (2014). Strategies to recruit minority persons: a systematic review. *Journal of Immigrant and Minority Health*.

[20] European Blood Alliance (2013). *MIMI Project—Action Plan for Minority Recruitment*.

[21] Australian Red Cross Blood Service, 2016, http://donateblood.com.au 10.5694/j.1326-5377.1997.tb123213.x 9152335

[22] New Zealand Blood Service, 2017, http://nzblood.co.nz

[23] Canadian Blood Services, 2017, http://blood.ca

[24] ADRP An International Division of America’s Blood Centers, 2017, https://www.adrp.org/

[25] America’s Blood Centers, 2012, http://americasblood.org/

[26] European Blood Alliance, 2017, https://europeanbloodalliance.eu

[27] World Bank (2017). World bank country and lending groups 2017. https://datahelpdesk.worldbank.org/knowledgebase/articles/906519-world-bank-country-and-lending-groups.

[28] Nance S. T. (2009). How to find, recruit and maintain rare blood donors. *Current Opinion in Hematology*.

[29] Shaz B. H. (2012). Minority donation in the United States. *ISBT Science Series*.

[30] Shaz B. H., Hillyer C. D. (2010). Minority donation in the United States: challenges and needs. *Current Opinion in Hematology*.

[31] Toni-Uebari T. K., Inusa B. P. (2009). The role of religious leaders and faith organisations in haemoglobinopathies: a review. *BMC Hematology*.

[32] National Health and Medical Research Council (2009). *NHMRC Levels of Evidence and Grades for Recommendations for Guideline Developers*.

[33] Critical Appraisal Skills Programme (CASP) (2017). *CASP Cohort Study Checklist*.

[34] Bachegowda L. S., Timm B., Dasgupta P. (2017). Impact of predictive scoring model and e-mail messages on African American blood donors. *Transfusion*.

[35] Price C. L., Boyd J. H., Watkins A. R., Fleming F., DeBaun M. R. (2006). Mailing of a sickle cell disease educational packet increases blood donors within an African American community. *Transfusion*.

[36] Robbins M. L., Paiva A. L., Amoyal N. R. (2015). Acceptability and feasibility of a culturally tailored internet-delivered intervention to promote blood donation in blacks. *Health Promotion Practice*.

[37] Sutton A. L. (2017). *A Targeted Approach to Increasing the African American Blood Donor Pool*.

[38] Charbonneau J., Daigneault S. (2016). Engaging ethnic minority blood donors. *ISBT Science Series*.

[39] Charbonneau J., Tran N. Y.-L. (2015). The paradoxical situation of blood donation in the Haitian-Quebec community. *Canadian Ethnic Studies*.

[40] Frye V., Caltabiano M., Kessler D. A. (2014). Evaluating a program to increase blood donation among racial and ethnic minority communities in New York city. *Transfusion*.

[41] Grassineau D., Papa K., Ducourneau A., Duboz P., Boëtsch G., Chiaroni J. (2007). Improving minority blood donation: anthropologic approach in a migrant community. *Transfusion*.

[42] Price C. L., Johnson M. T., Lindsay T., Dalton D., DeBaun M. R. (2009). The sickle cell sabbath: a community program increases first-time blood donors in the African American faith community. *Transfusion*.

[43] Moher D., Liberati A., Tetzlaff J., Altman D. G., The Prisma Group (2009). Preferred reporting items for systematic reviews and meta-analyses: the prisma statement. *PLoS Medicine*.

[44] Buntin M. B., Burke M. F., Hoaglin M. C., Blumenthal D. (2011). The benefits of health information technology: a review of the recent literature shows predominantly positive results. *Health Affairs*.

[45] Netto G., Bhopal R., Lederle N., Khatoon J., Jackson A. (2010). How can health promotion interventions be adapted for minority ethnic communities? Five principles for guiding the development of behavioural interventions. *Health Promotion International*.

[46] Nápoles-Springer A. M., Ortíz C., O’Brien H., Díaz-Méndez M. (2009). Developing a culturally competent peer support intervention for Spanish-speaking Latinas with breast cancer. *Journal of Immigrant and Minority Health*.

[47] James A. B., Schreiber G. B., Hillyer C. D., Shaz B. H. (2013). Blood donations motivators and barriers: a descriptive study of African American and white voters. *Transfusion and Apheresis Science*.

[48] Polonsky M. J., Renzaho A. M. N., Ferdous A. S., McQuilten Z. (2013). African culturally and linguistically diverse communities’ blood donation intentions in Australia: integrating knowledge into the theory of planned behavior. *Transfusion*.

[49] Boenigk S., Mews M., de Kort W. (2015). Missing minorities: explaining low migrant blood donation participation and developing recruitment tactics. *Voluntas: International Journal of Voluntary and Nonprofit Organizations*.

[50] Anand R., Cosulich R. (2016). Ethnic minority blood donation-challenges and needs. *Transfusion Medicine*.

