# Supplementary material for: Interventions to Increase Blood Donation among Ethnic/Racial Minorities: A Systematic Review
Source: J Environ Public Health. 2019 Apr 15;2019:6810959. doi: 10.1155/2019/6810959 (PMC6501160; doi:10.1155/2019/6810959)
Supplement: Supplementary Materials — Supplementary Material file includes Tables S1 (search terms), S2 (criteria for critical appraisal), and S3 (reasons for exclusion of full-text articles). [file 6810959.f1.pdf]

S1. Search terms (formatted for Medline via Ovid)

#1	Blood Donors/ OR 'Blood donation'.tw. OR 'Blood donor'.tw. OR 'Blood bank'.tw.
#2	Minority Groups/ OR Ethnic Groups/ OR Refugees/ OR African Americans/ OR Hispanic Americans/ OR Minority.tw. OR Ethnic.tw. OR Migrant.tw. OR Refugee.tw. OR Indigenous.tw. OR African.tw. OR Asian.tw. OR Hispanic.tw. OR Latino.tw. OR Haitian.tw. OR Chinese.tw.
#3	#1 AND #2
#4	#3 NOT (Comment/ OR Letter/ OR Editorial/ OR News/)

Search string for Google Scholar: Blood donor ethnic minority

S2: Criteria for critical appraisal

**NHMRC level of evidence**	
I	A systematic review of level II studies
II	A randomised controlled trial
III-1	A pseudorandomised controlled trial (i.e. alternate allocation or other method)
III-2	A comparative study with concurrent controls: ▪ Non-randomised, experimental trial<br>▪ Cohort study<br>▪ Case-control study<br>▪ Interrupted time series with a control group
III-3	A comparative study without concurrent controls:<br>▪ Historical control study<br>▪ Two or more single arm study<br>▪ Interrupted time series without a parallel control group
IV	Case series with either post-test or pre-test/post-test outcomes
**NHMRC Magnitude of effect**	
Low	Difference is statistically significant AND the confidence interval does not include any clinically important effects<br>OR<br>Difference is not statistically significant AND the range of estimates defined by the confidence interval includes clinically important effects
Medium	Difference is statistically significant AND the point estimate of effect is clinically important but the confidence interval includes some clinically unimportant effects
High	Difference is statistically significant AND there is a clinically important benefit for the full range of estimates defined by the confidence interval
**CASP risk of bias appraisal**	
1	Did the study address a clearly focussed issue?
2	Were the participants recruited in an acceptable way?
3	Was the exposure accurately measured to minimise bias?
4	Was the outcome accurately measured to minimise bias?
5	Have the authors identified all important confounding factors?
6	Have they taken account of the confounding factors in the design and/or analysis?
7	Was the follow up of participants complete enough?
8	Was the follow up of participants long enough?
9	Any other bias concerns

S3: Reasons for exclusion of full text articles

**Reason for exclusion**	**# of articles**
Did not evaluate an intervention	95
Was not conducted in a High Income country	41
Did not relate to blood donation	13
Only available as a conference abstract	10
Reported on blood donation for research, not transfusion	8
Did not report outcomes	3
Did not include ethnic/racial minority participants	4
Had no English full-text version available	2
Did not include adults as participants	1
